# Supplementary material for: Spatial Distribution and Dietary Risk Assessment of Aflatoxins in Raw Milk and Dairy Feedstuff Samples from Different Climate Zones in China
Source: Toxins (Basel). 2025 Jan 16;17(1):41. doi: 10.3390/toxins17010041 (PMC11769556; doi:10.3390/toxins17010041)
Supplement: Supplementary file 1 [file toxins-17-00041-s001.zip › Table S1.pdf]

Table S1. MS/MS acquisition parameters for aflatoxins

Mycotoxin	Precursor ion (m/z)	Quantitative ion (m/z)	Collision energy (V)	Qualitative ion (m/z)	Collision energy (V)
AFTB1	313	285	22	241	38
$^{13}C_{17}$-AFTB1	330	255	23	301	35
AFM1	329	273	23	259	23
$^{13}C_{17}$-AFM1	346	317	23	288	24
