# Supplementary figures and images for: Spatial Distribution and Dietary Risk Assessment of Aflatoxins in Raw Milk and Dairy Feedstuff Samples from Different Climate Zones in China
Source: Toxins (Basel). 2025 Jan 16;17(1):41. doi: 10.3390/toxins17010041 (PMC11769556; doi:10.3390/toxins17010041)

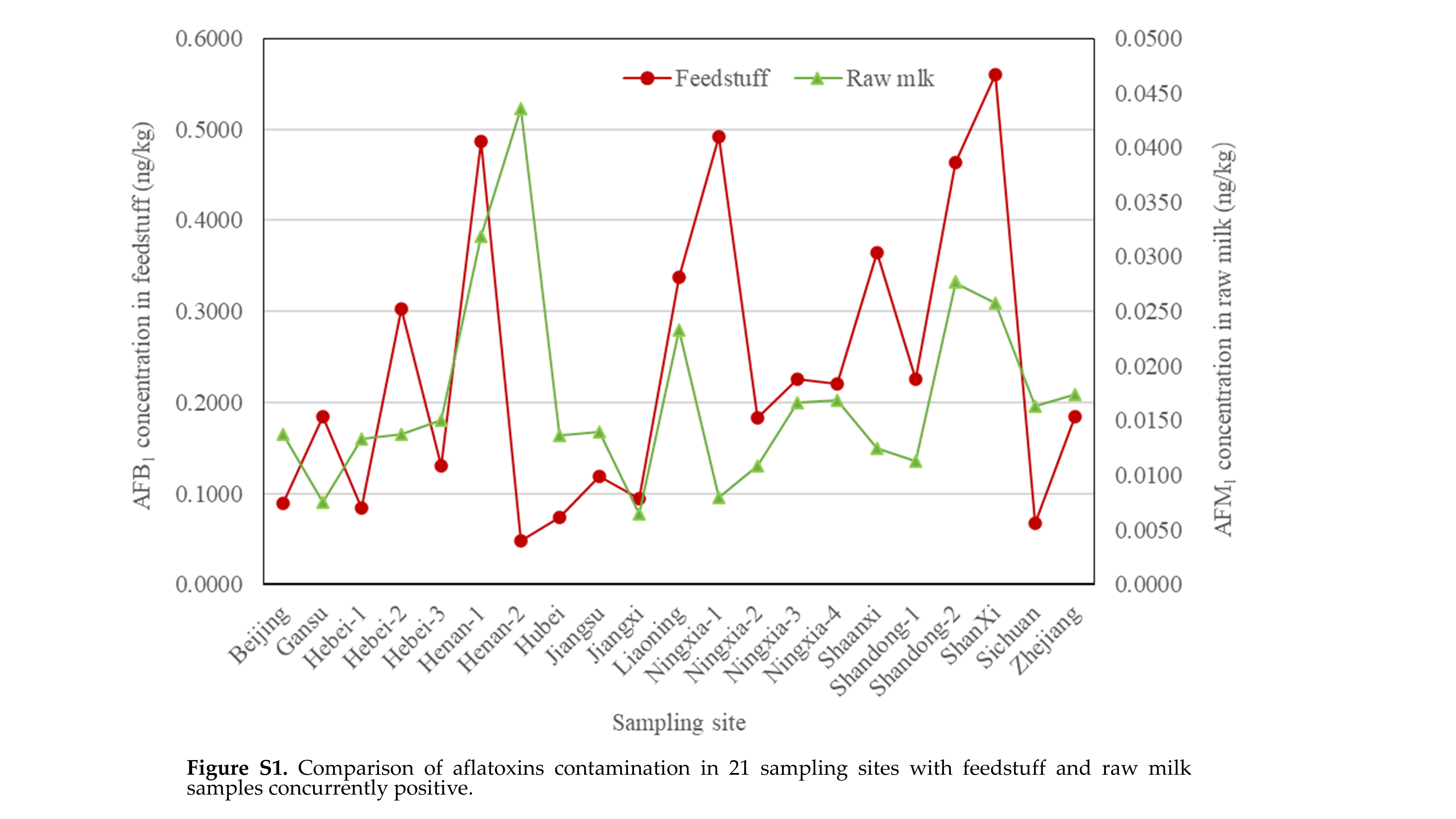

Supplement: Supplementary file 1 [file toxins-17-00041-s001.zip › Figure S1.tif]

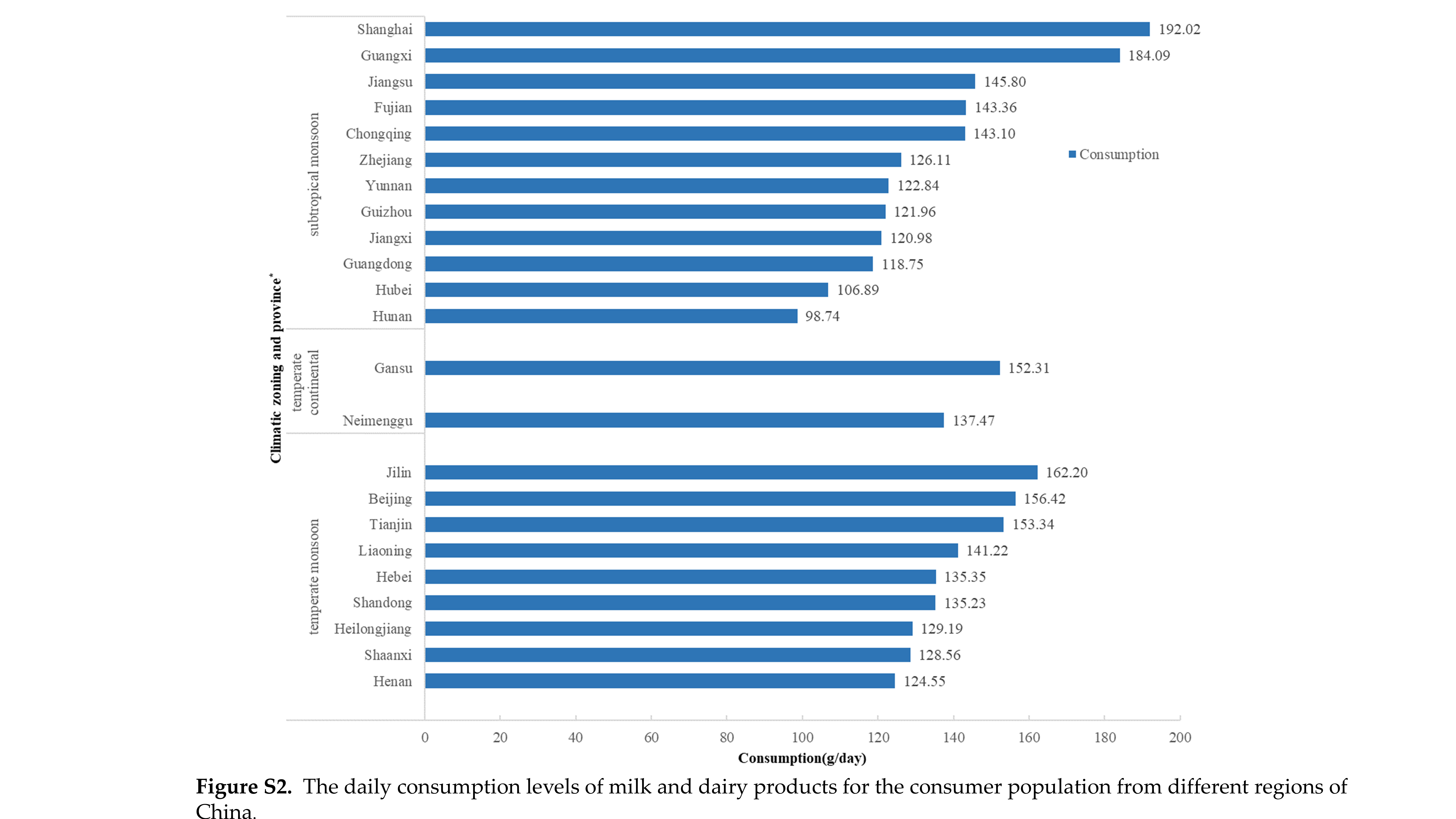

Supplement: Supplementary file 1 [file toxins-17-00041-s001.zip › Figure S2.tif]

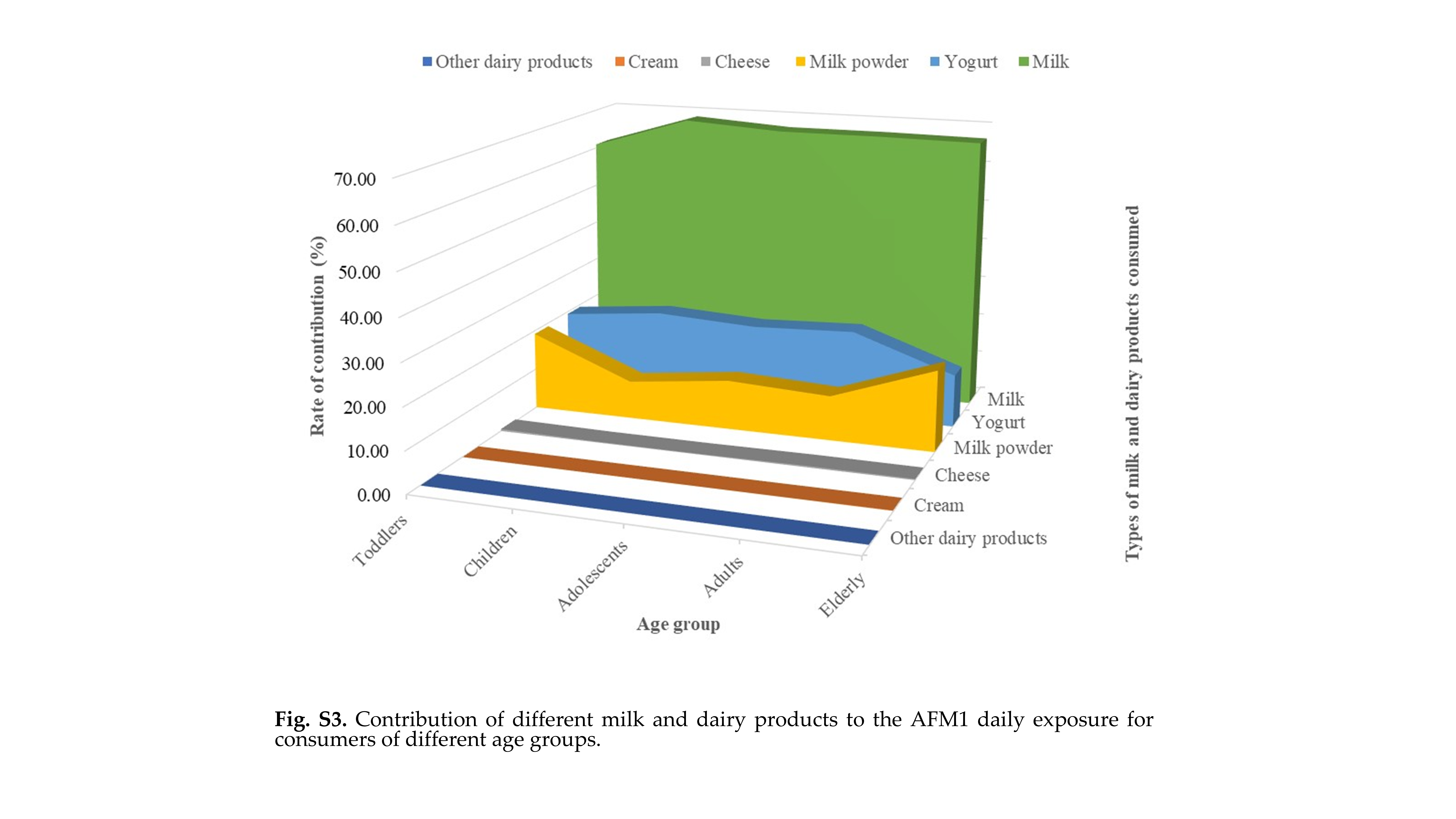

Supplement: Supplementary file 1 [file toxins-17-00041-s001.zip › Figure S3.tif]

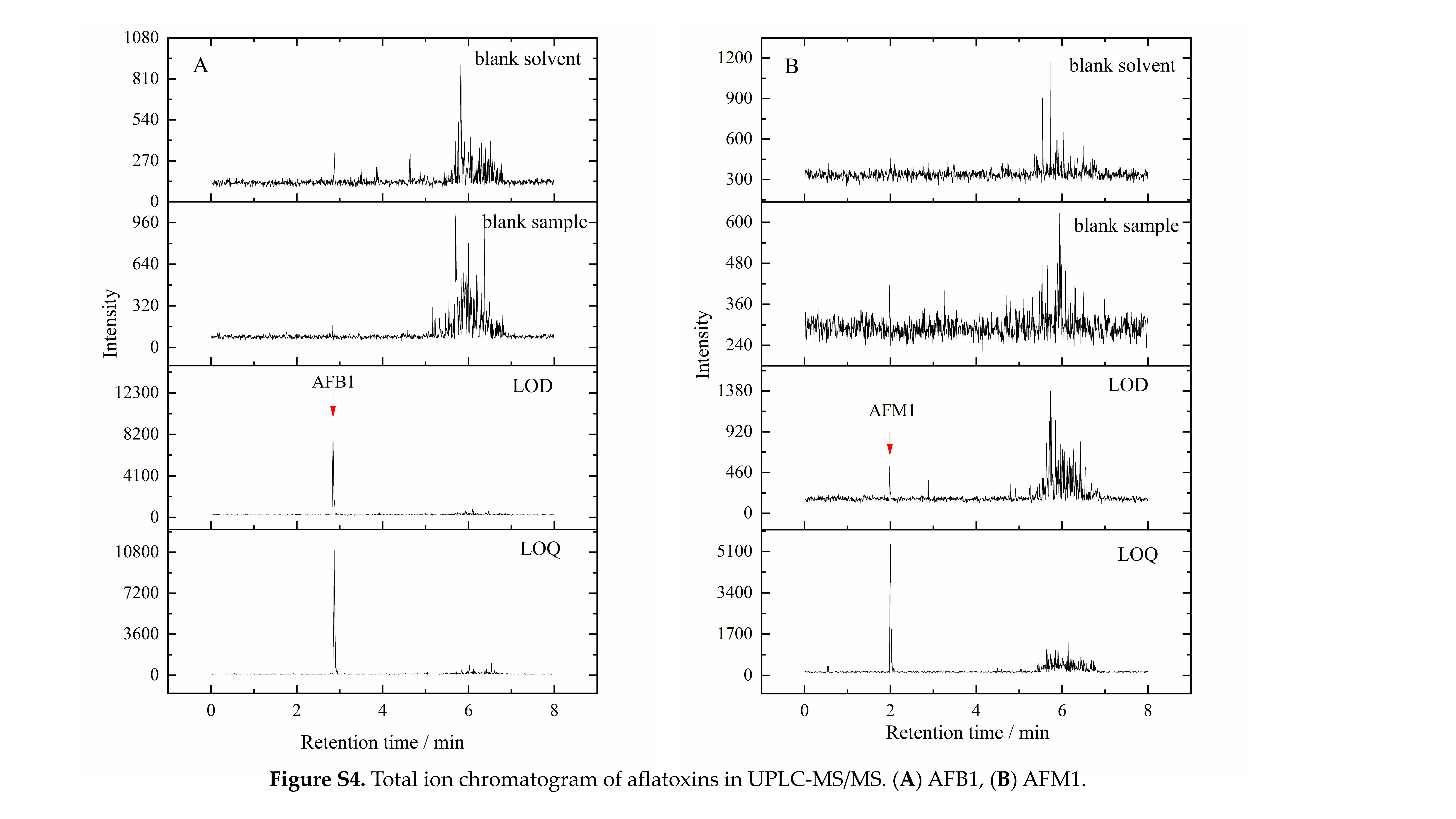

Supplement: Supplementary file 1 [file toxins-17-00041-s001.zip › Figure S4.tif]
